# Analysis of Volatile Compounds from Different Parts of *Houttuynia cordata* Thunb.

**DOI:** 10.3390/molecules27248893

**Published:** 2022-12-14

**Authors:** Chen-Hsiang Lin, Louis Kuoping Chao, Li-Yun Lin, Chin-Sheng Wu, Lee-Ping Chu, Chien-Hsueh Huang, Hsin-Chun Chen

**Affiliations:** 1 Taoyuan District Agricultural Research and Extension Station, Council of Agriculture, Executive Yuan, Taoyuan 327, Taiwan; 2 Department of Cosmeceutics, China Medical University, Taichung 406, Taiwan; 3 Department of Food Science and Technology, Hungkuang University, Taichung 433, Taiwan; 4 Department of Pharmacy, China Medical University Hospital, Taichung 404, Taiwan; 5 Department of Orthopedics, China Medical University Hospital, Taichung 404, Taiwan

**Keywords:** *Houttuynia cordata* Thunb., essential oil, gas chromatography-mass spectrometry (GC-MS), headspace solid-phase microextraction (HS-SPME)

## Abstract

*Houttuynia cordata* Thunb. is a medicinal and edible plant that has been commonly used in traditional Chinese medicine since ancient times. This study used headspace solid-phase microextraction (HS-SPME) and direct injection, combined with gas chromatography (GC) and gas chromatography-mass spectrometry (GC-MS), to identify the volatile compounds in *H. cordata.* Extraction from different parts of the plant using different extraction techniques for the identification of volatile compounds were determined. A total of 93 volatile components were analyzed in the leaves, stems, rhizomes, and whole plant samples of *H. cordata*. The leaves contained more (*Z*)-3-hexenal, β-myrcene, (*Z*)-β-ocimene, and (4*E*,6*E*)-*allo*-ocimene; the stems contained more geranyl acetate and nerolidol; and rhizomes contained more α-pinene, β-pinene, limonene, 2-undecanone, and decanoyl acetaldehyde. Among them, the essential oil extracted by HS-SPME could produce more monoterpenes, while direct injection could obtain higher contents of aliphatic ketones, terpene esters, sesquiterpenes, and was more conducive to the extraction of 2-undecanone and decanoyl acetaldehyde.

## 1. Introduction

*Houttuynia cordata* Thunb., belonging to Saururaceae, is a medicinal and edible perennial herb native to China, Japan, and Taiwan [1]. *H. cordata* is rich in nutrients and contains a variety of vitamins, amino acids, and trace elements, such as zinc, potassium, and copper [2]. The physiologically active substances of *H. cordata* include essential oils, steroids, and flavonoids [3], which have many pharmacological properties, including antibacterial, antiviral, anti-inflammatory, antioxidant, and anticancer effects. Many studies have been conducted on its active components and pharmacological properties [4,5,6,7,8].

Essential oils, secondary metabolites of plants, are industrially important natural products [9]. Essential oils were utilized in pharmaceutical and other related medical, and the amount of published evidence on aromatherapy and essential oils has gradually increased [10,11]. The essential oil components of *H. cordata* include decanoyl acetaldehyde, 2-undecanone, β-myrcene, decanal, and *trans*-caryophyllene [1]. Because the essential oil compounds of *H. cordata* will affect its pharmacological effects, analyses of the volatile components are also used to determine plant quality [12].

To date, there have been many studies on the volatile components of *H. cordata*. Kosuge [13] used steam distillation to extract essential oils from *H. cordata* and isolated decanoyl acetaldehyde, which has an antibacterial effect and is known to cause the unique stinking smell of *H. cordata* [14]. However, this component is easily oxidized into 2-undecanone during distillation and storage [15]. Both are important volatile components of *H. cordata* [14,16]. Yang et al. [17] analyzed 25 volatile compounds in *H. cordata* by GC-MS, including α-pinene, camphene, β-pinene, β-myrcene, (+)-limonene, γ-terpinene, decanal, linalool, β-caryophyllene, and 2-undecanone. Asakawa et al. [18] analyzed volatile compounds in different parts of the *H. cordata* plant. The study indicated that the main component of all parts analyzed was 4-tricancanone, and β-myrcene was the main monoterpene in the flowers, leaves, and stems, while the main monoterpene in the rhizomes and roots was β-pinene, and 1-decanal was the main polyketide in leaves and stems. Xu et al. [19] analyzed monoterpenes in the essential oils of three *H. cordata* accessions, and the results showed that the number and content of monoterpenes were different in different plant parts and different accessions.

In this study, extraction from different parts of the *H. cordata* plant, and different extraction methods on the volatile components. The results of this study can be used as a reference for the extraction and utilization of *H. cordata* in the future.

## 2. Results and Discussion

### 2.1. Analysis of Volatile Compounds from Different Parts of H. cordata

**Figure 1:**
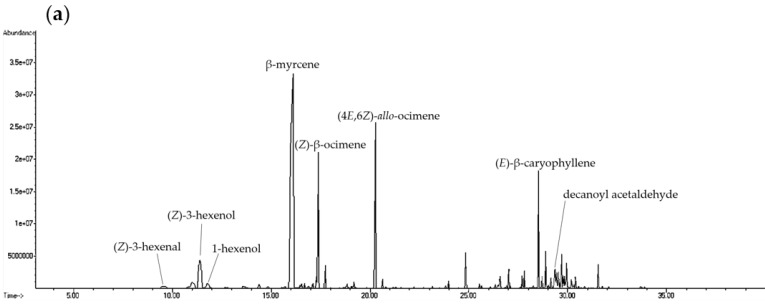

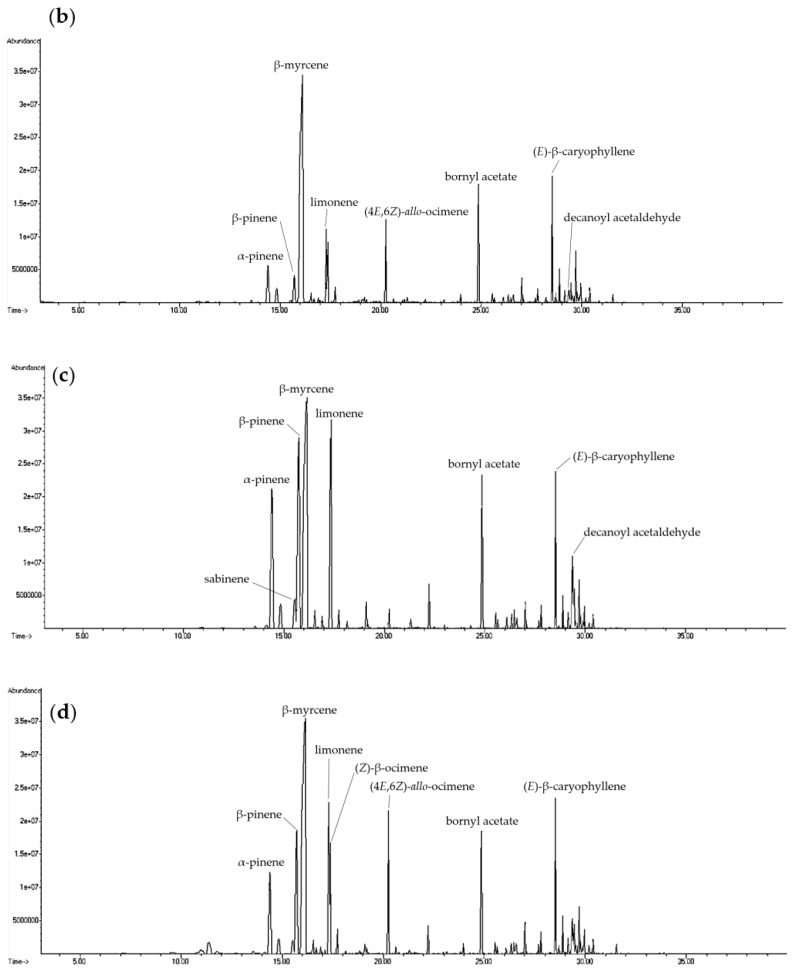
Ion chromatogram of volatile compounds from different parts of fresh *H. cordata* analyzed by HS-SPME: (**a**) leaf; (**b**) stem; (**c**) rhizome; and (**d**) whole plant.

**Figure 2:**
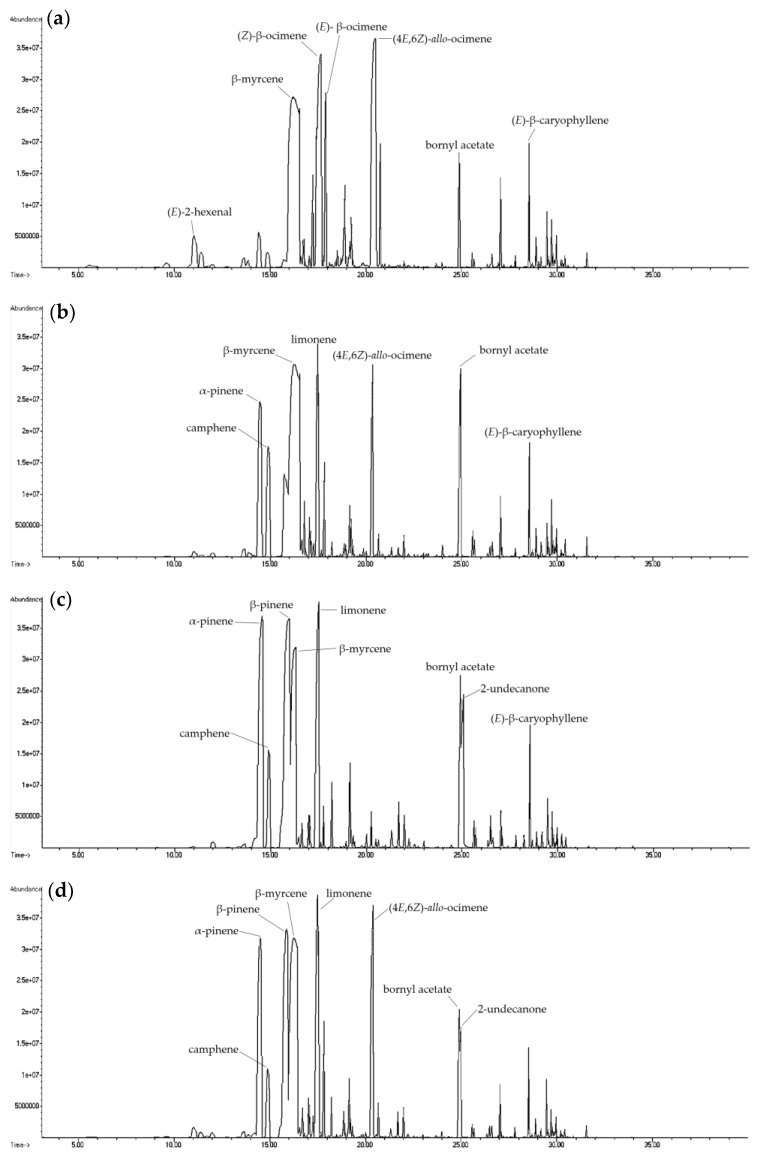
Ion chromatogram of volatile compounds in essential oil from different parts of fresh *H. cordata* analyzed by HS-SPME: (**a**) leaf; (**b**) stem; (**c**) rhizome; and (**d**) whole plant.

**Figure 3:**
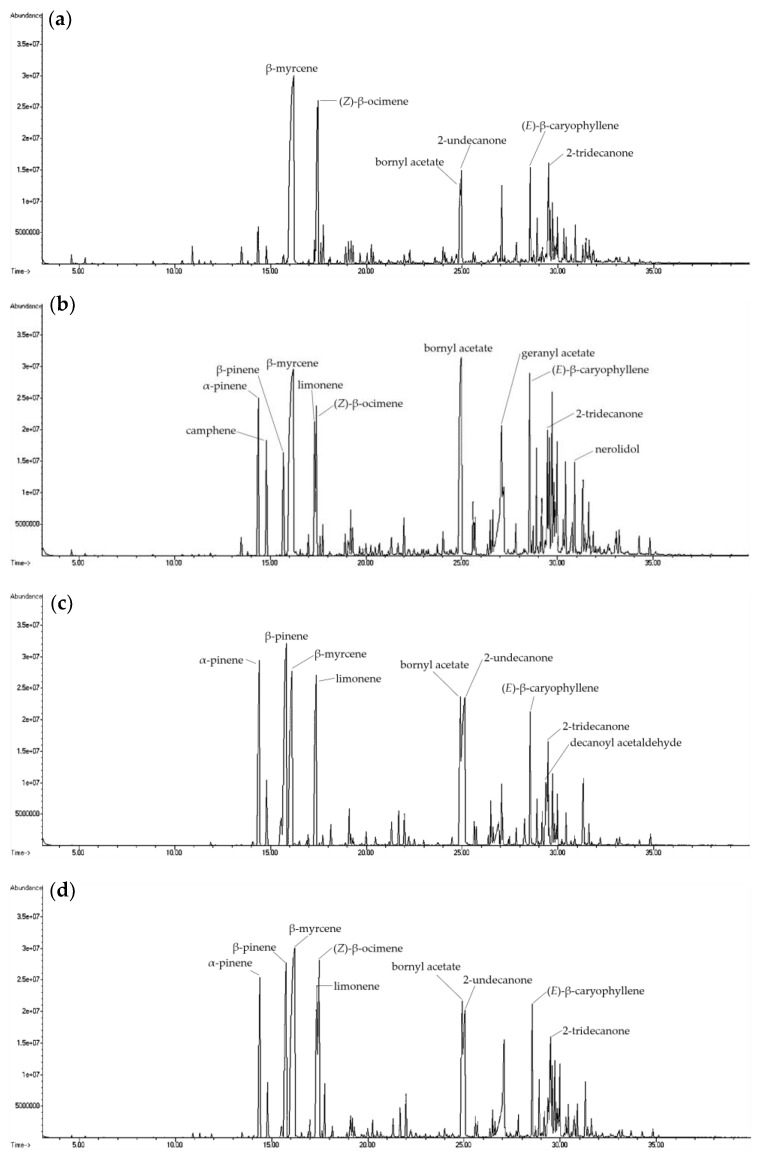
Ion chromatogram of volatile compounds in essential oil from different parts of fresh *H. cordata* analyzed by direct injection: (**a**) leaf; (**b**) stem; (**c**) rhizome; and (**d**) whole plant.

The leaves, stems, rhizomes, and whole plants of fresh *H. cordata* were analyzed for differences in volatile compounds (Figure 1, Figure 2 and Figure 3). Fresh plants and essential oils were analyzed using HS-SPME and direct injection. A total of 91 volatile components were identified in the *H. cordata* samples. The yield of essential oils was 0.09% (leaves), 0.02% (stems), 0.04% (rhizomes), and 0.04% (whole plants). Chen et al. [20] analyzed *Angelica acutiloba* essential oil and found that the highest content of essential oils was in the leaves. The main compounds from different parts were not the same, and the overall components and contents were different. In addition, the year-to-year yields of essential oil were slightly different, which may be due to differences in cultivation and climatic conditions [21].

**Figure 4:**
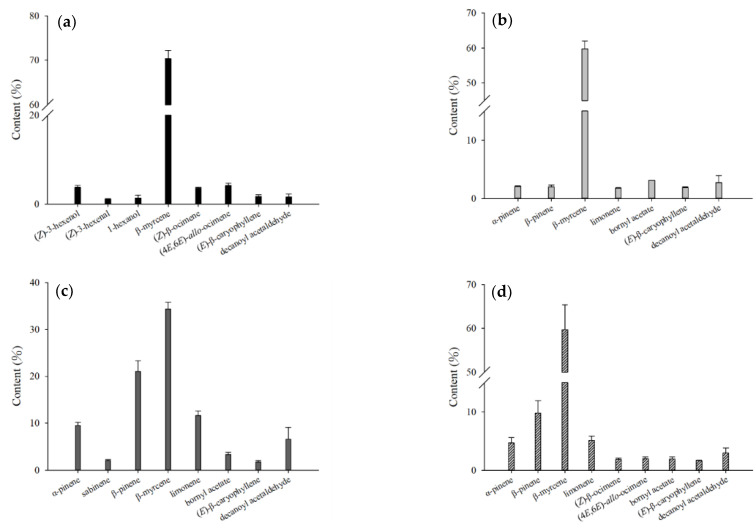
Comparison of the contents of the major volatile compounds from different parts of fresh *H. cordata* analyzed by HS-SPME: (**a**) leaf; (**b**) stem; (**c**) rhizome; and (**d**) whole plant.

Analysis of fresh *H. cordata* samples using HS-SPME found that the main volatile compounds in the leaves were (*Z*)-3-hexenal, (*Z*)-3-hexenol, 1-hexenol, β-myrcene, (*Z*)-β-ocimene, (4*E*,6*E*)-*allo*-ocimene, (*E*)-β-caryophyllene, and decanoyl acetaldehyde. The main volatile compounds in the stem were α-pinene, β-pinene, β-myrcene, limonene, bornyl acetate, (*E*)-β-caryophyllene, and decanoyl acetaldehyde. The main volatile compounds in the rhizomes were α-pinene, sabinene, β-pinene, β-myrcene, limonene, bornyl acetate, (*E*)-β-caryophyllene, and decanoyl acetaldehyde. The main volatile compounds in the whole plant were α-pinene, β-pinene, β-myrcene, limonene, (*Z*)-β-ocimene, (4*E*,6*E*)-*allo*-ocimene, bornyl acetate, (*E*)-β-caryophyllene, and decanoyl acetaldehyde. β-myrcene had the highest content within all samples (Figure 4). Comparing the composition of leaves, stems, and rhizomes, the content of (*Z*)-3-hexenal, (*Z*)-3-hexenol, 1-hexenol, β-myrcene, (*Z*)-β-ocimene, and (4*E*,6*E*)-*allo*-ocimene in leaves was significantly higher than in the other parts of the plant. The rhizomes contained the highest content of α-pinene, sabinene, β-pinene, limonene, bornyl acetate, and decanoyl acetaldehyde. The stem had no components that were significantly higher than those of the leaves and rhizomes (Table 1).

**Figure 5:**
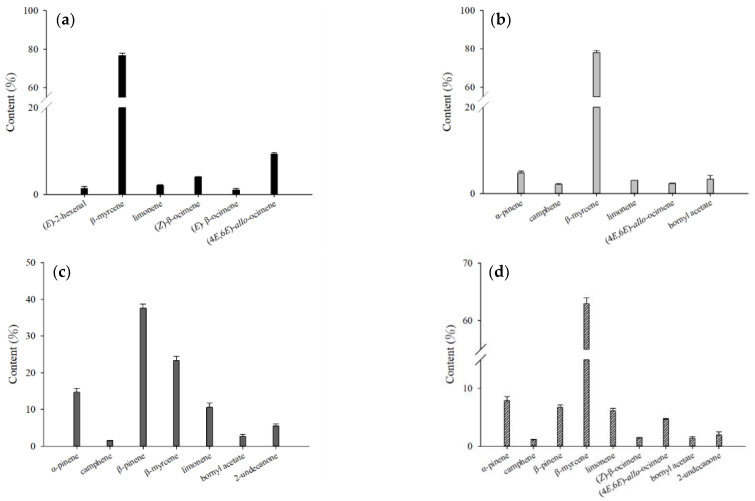
Comparison of the contents of the major volatile compounds in essential oil from different parts of fresh *H. cordata* analyzed by HS-SPME: (**a**) leaf; (**b**) stem; (**c**) rhizome; and (**d**) whole plant.

The essential oils from different parts of *H. cordata* were extracted by steam distillation and then analyzed by HS-SPME and direct injections. The results showed that the main volatile components of essential oil from the leaves, as analyzed by HS-SPME, were (*E*)-2-hexenal, β-myrcene, limonene, (*Z*)-β-ocimene, (*E*)-β-ocimene, and (4*E*,6*E*)-*allo*-ocimene; in the stems were α-pinene, camphene, β-myrcene, limonene, (4*E*,6*E*)-*allo*-ocimene, and bornyl acetate; and in the rhizomes were α-pinene, camphene, β-pinene, β-myrcene, limonene, bornyl acetate, and 2-undecanone. In the whole plant were α-pinene, camphene, β-pinene, β-myrcene, limonene, (*Z*)-β-ocimene, (4*E*,6*E*)-*allo*-ocimene, bornyl acetate, and 2-undecanone (Figure 5). Among the three parts, the leaves contained the highest content of (*Z*)-3-hexenal, (*E*)-2-hexenal, (*Z*)-β-ocimene, (*E*)-β-ocimene, and (4*E*,6*E*)-*allo*-ocimene; the stem had the highest content of camphene, β-myrcene, and bornyl acetate, and the rhizome contained the highest content of α-pinene, β-pinene, limonene, and 2-undecanone.

**Figure 6:**
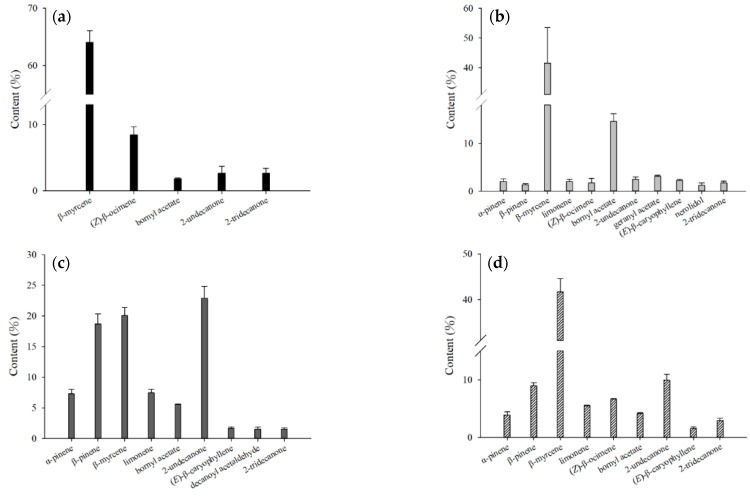
Comparison of the contents of the major volatile compounds in essential oil from different parts of fresh *H. cordata* analyzed by direct injection: (**a**) leaf; (**b**) stem; (**c**) rhizome; and (**d**) whole plant.

The essential oils of leaves analyzed by direct injection were mainly composed of β-myrcene, (Z)-β-ocimene, bornyl acetate, 2-undecanone and 2-tridecanone; in the stems were α-pinene, β-pinene, β-myrcene, limonene, (Z)-β-ocimene, bornyl acetate, 2-undecanone, geranyl acetate, (*E*)-β-caryophyllene, nerolidol, and 2-tridecanone; in the rhizomes were α-pinene, β-pinene, β-myrcene, limonene, bornyl acetate, 2-undecanone, (*E*)-β-caryophyllene, decanoyl acetaldehyde, and 2-tridecanone; and in the whole plant were α-pinene, β-pinene, β-myrcene, limonene, (Z)-β-ocimene, bornyl acetate, 2-undecanone, (*E*)-β-caryophyllene, and 2-tridecanone (Figure 6). After comparing the components of leaves, stems, and rhizomes, the leaves contained the highest content of β-myrcene, (Z)-β-ocimene, and 2-tridecanone; the stems had the highest content of bornyl acetate, geranyl acetate, (*E*)-β-caryophyllene, and nerolidol; and the rhizomes contained the highest amounts of α-pinene, β-pinene, limonene, 2-undecanone, and decanoyl acetaldehyde.

Comparing the results from different parts of fresh plants with HS-SPME, there was more (*Z*)-3-hexenol, 1-hexanol, (*E*)-2-hexenal, (2*E*,4*E*)-hexadienal, α-cubebene, and (*Z*,*E*)-α-farnesene in the leaves than in other parts. Linalool, perilla alcohol, nerolidol, and caryophyllene oxide only appeared in the stems, and n-nonanyl acetate, p-cymenene, α-thujene, γ-terpinene, terpinene-4-ol, α-terpinyl acetate, and neryl acetate were only identified in rhizomes.

When comparing the results of the essential oils from the three parts analyzed by HS-SPME, the components that were only identified in the leaves included (*Z*)-3-hexenal, β-terpinene, 4-ethyl-1,2-dimethylbenzene, α-copaene, alloaromadendrene, (*Z*,*E*)-α-farnesene, β-selinene, β-bisabolene, cadina-1,4-diene, and β-damascenone. The only compound identified in the stems was borneol. The components that were only identified in rhizomes included 1-nonanol, 1-decanol, hexanal, α-campholenal, decanoyl acetaldehyde, 2-nonanone, 3-dodecanone, α-thujene, δ-3-carene, geranial, α-terpinyl acetate, neryl acetate, carvone, (*E*)-limonene oxide, and caryophyllene oxide.

The results of analyzing essential oils by direct injection showed that compounds only identified in the leaves included 3-methyl-2-buten-1-ol, (*Z*)-3-hexenal, hexanal, (2*E*,4*E*)-hexadienal, α-cubebene, and β-damascenone. The compounds only appearing in the stems were dodecanal, methyl salicylate, and borneol. The compounds only found in the rhizomes were 1-nonanol, 1-decanol, α-campholenal, decanal, 2-nonanone, 3-dodecanone, α-fenchene, terpinen-4-ol, geranial, fenchyl acetate, (2*E*,6*E*)-farnesyl acetate, carvone, and (*E*)-limonene oxide.

Asakawa et al. [18] analyzed the volatile components of different parts of *H. cordata* and showed that the main component of each part was 4-tricancanone. The main monoterpene in rhizomes and roots was β-pinene, while in flowers, leaves, and stems, it was β-myrcene. 1-decanal is the main polyketide compound in leaves and stems. Haghighi et al. [27] studied the effects of ecotypes and different plant parts (leaves, flowers, and fruits) on essential oil from *Vitex pseudo-negundo*. The results showed that there were significant differences in the yield and chemical characteristics of the essential oils in different plant parts. Zribi et al. [28] analyzed the volatile components and essential oils of Tunisian *Borago officinalis* L. and showed that the main components of different parts of this plant differed; octanal was the main component in the flowers, while in leaves, it was nonanal.

### 2.2. Comparison of Different Extractions

Comparing HS-SPME of fresh plants, HS-SPME of essential oil, and direct injection of essential oil, HS-SPME from fresh plants produced the smallest number of volatile compounds, followed by HS-SPME of essential oil, while direct injection of essential oil produced the most. Analysis of fresh plants by HS-SPME identified aliphatic alcohols and aliphatic aldehydes of low molecular weight, while analysis of essential oils by HS-SPME identified the highest content of monoterpenes. Direct injection of essential oils could identify more aliphatic ketones, sesquiterpenes, and terpene esters (Table 2).

Farag and Wessjohann [29] compared the volatile compound profiles of *Glycyrrhiza glabra* L. roots extracted by SPME and steam distillation. The results showed that SPME could easily extract several small molecular weight monoterpenes, while more compounds could be identified in the essential oils extracted by steam distillation, including the volatiles generated by chemical reactions during the heating process. Peng et al. [30] analyzed the volatile components of kumquat (*Fortunella margarita* Swingle) and showed that HS-SPME/GC could identify a higher proportion of monoterpenes but fewer sesquiterpenes than that by DI/GC. Gao et al. [31] compared different extractions to volatile components of Pu-erh ripe tea and observed that HS-SPME was beneficial for the extraction of highly volatile compounds, such as low molecular weight alcohols, aldehydes, ketones, and hydrocarbons.

Yang et al. [32] compared HS-SPME with conventional extraction in the analysis of *Melia azedarach*, and Kung et al. [33] analyzed *Platostoma palustre* (Blume) and pointed out that HS-SPME is a powerful analytical tool that can complement traditional methods. Overall, the results of these three methods showed all have high monoterpene content. Direct injection can be used to analyze more classes of volatile compounds, especially the larger molecular weight components, including important components of *H. cordata* such as 2-undecanone, and decanoyl acetaldehyde. 

*H. cordata* has long been used as an edible vegetable and in traditional medicine [2]. Due to its pharmacological properties, it has been gradually applied in many fields, such as medicine, health food, preservatives and cosmetics, with great potential for development.

## 3. Materials and Methods

### 3.1. Plant Materials

*H. cordata* used in this study was provided by the Taoyuan District Agricultural Research and Extension Station, Council of Agriculture, Executive Yuan (24°57′09.3″ N 121°01′42.4″ E, altitude = 39 m). The experiment was designed with three replicates. Three parts (leaves, stems, and rhizomes) and fresh whole *H. cordata* plants were collected for experiments. Precisely 3 kg of voucher specimens for each batch were dried and deposited. A herbarium sample (No. TY 1406) was lodged at the Flavor and Fragrance Research Laboratory at China Medical University, Taiwan.

### 3.2. Analytical Methods

#### 3.2.1. Extraction of Volatile Compounds from Fresh *H. cordata* by HS-SPME

After homogenizing (Electrolux, ECG120S) the samples for 15 s, 3 g were weighed and placed in a 22 mL cylindrical glass bottle (Supelco Co., No. 27170, Bellafonte, PA, USA) then sealed with Teflon rubber pad. The fiber was coated with 50/30 μm DVB/CAR/PDMS (Supelco) and extracted for 30 min. The extraction temperature was room temperature. After the extraction was complete, the fiber was inserted into the inlet of the GC or GC-MS and desorbed for 20 min. This experiment was repeated in triplicate.

#### 3.2.2. Extraction of Essential Oil from *H. cordata*

A fresh *H. cordata* sample (600 g) was washed with clean water, and then 1800 mL of distilled water was added to homogenize (TATUNG, TJC-2200) for 30 s. The homogenate was then placed in a 5 L round-bottom flask for steam distillation. The extraction time was 3 h, and the extract was stored at 4 °C until analysis. The experiment was repeated in triplicates. The yields of essential oils from different parts of *H. cordata* were 0.09% (leaves), 0.02% (stems), 0.04% (rhizomes), and 0.04% (whole plants).

#### 3.2.3. Gas Chromatography-Flame Ionization Detector (GC-FID)

GC was performed with an Agilent Model 7890A GC (Santa Clara, CA, USA), with a 60 m × 0.25 mm id Agilent DB-1 fused-silica capillary non-polar column with a film thickness of 0.25 μm; the HS-SPME injection mode was splitless, and the injection mode of direct injection was split. The GC heating conditions were as follows: the initial temperature was maintained at 40 °C for 1 min, then raised to 150 °C at 5 °C/min, maintained for 1 min, then raised to 200 °C at 10 °C/min and maintained for 11 min. The inlet temperature was 250 °C, the detector temperature was 300 °C, and a flame ionization detector (FID) was used for detection. The carrier gas was nitrogen at a flow rate of 1 mL/min.

#### 3.2.4. Gas Chromatography-Mass Spectrometry (GC-MS)

GC-MS was conducted with an Agilent Model 5977A quadrupole mass spectrometer (Mass Selective Detector, MSD) coupled to an Agilent Model 7890B GC (Palo Alto, CA, USA). The operating conditions and column were the same as in Section 3.2.3. The carrier gas was helium, the ion source temperature of the MSD was 230 °C, and the electron energy was 70 eV. The transfer line was set at 250 °C. The mass range was 30–350 m/z. The quadrupole temperature was 150 °C. The mass spectra data were compared and judged using the Wiley 7N mass spectrum library.

#### 3.2.5. Analysis of Essential Oil from *H. cordata*

For GC, 1 µL of essential oil was injected, while 0.5 µL was injected for GC-MS. For HS-SPME, 0.1 mL of the essential oil was added to a 4 mL cylindrical glass bottle (Supelco Co., No. 27136) with a Teflon rubber pad. Additionally, 50/30 μm DVB/CAR/PDMS fiber was used for extraction for 5 min, and the extraction temperature was room temperature. The fiber was then inserted into the inlet of the GC or GC-MS. All experiments were performed in triplicates.

#### 3.2.6. Retention Index (RI) Comparison

The GC retention index of the volatile components in this experiment was based on a mixture of C_5_–C_25_ *n*-alkane standards (Sigma-Aldrich, St. Louis, MO, USA) and the GC retention time was used as a reference under the same conditions. The RI was calculated according to the method described by Curvers et al. in reference [34].

#### 3.2.7. Relative Percentage Calculation

After volatile components were identified, the percentage composition was calculated using the peak area normalization measurements. The formula is as follows:

$$\frac{\mathrm{volatile}\;\mathrm{component}\;\mathrm{peak}\;\mathrm{area}}{\mathrm{total}\;\mathrm{peak}\;\mathrm{areas}}\times 100\%$$

In addition to the volatile compounds of the sample, HS-SPME will also adsorb the impurity of the bottle or any silicon-containing coating. The total percentage in the above tables did not reach 100%, due to deducted from these impurities.

## 4. Conclusions

The volatile compounds of *H. cordata* leaves, stems, rhizomes, and whole plants were compared. The leaves of *H. cordata* had the highest essential oil content, but the rhizomes had higher 2-undecanone and decanoyl acetaldehyde content. Therefore, using rhizomes as raw materials is beneficial for extracting key components of *H. cordata.* Additionally, HS-SPME and direct injection of essential oil are highly complementary. Together, they cover the full range of volatilities and trace components and provide relatively complete data on the volatile components of *H. cordata.*

## Figures and Tables

**Table 1:** Comparison of the content of volatile compounds from different parts of *H. cordata* and their essential oils, analyzed by HS-SPME and direct injection.

Compound	CAS Number	Molecular Formula	RI ^a^	RI ^b^	Relative Content (%) ^c^											
					HS-SPME				Essential Oil HS-SPME				Essential Oil Direct Injection			
					Leaf	Stem	Rhizome	Whole ^d^	Leaf	Stem	Rhizome	Whole	Leaf	Stem	Rhizome	Whole
***Aliphatic Alcohols***																
prenol	556-82-1	C_5_H_10_O	751.5	745									0.04 ± 0.01			
(*Z*)-3-hexenol	928-96-1	C_6_H_12_O	840	830	3.76 ± 0.45			0.47 ± 0.37	0.75 ± 0.07	0.08 ± 0.01		0.29 ± 0.06	0.08 ± 0.05	0.02 ± 0.01		0.07 ± 0.02
1-hexanol	111-27-3	C_6_H_14_O	854	849	1.38 ± 0.62			0.23 ± 0.09								
(*E*)-*p*-menth-2-en-1-ol	29803-81-4	C_10_H_18_O	1112	1101						0.02 ± 0.01	0.03 ± 0.01			0.14 ± 0.03	0.14 ± 0.01	0.11 ± 0.01
1-nonanol	143-08-8	C_9_H_20_O	1157	1147					<0.01		0.05 ± 0.04	0.01 ± 0.001			0.27 ± 0.10	0.02 ± 0.004
1-decanol	112-30-1	C_10_H_22_O	1257	1256							0.01 ± 0.002				0.03 ± 0.004	0.08 ± 0.01
***Aliphatic Aldehydes***																
(*Z*)-3-hexenal	6789-80-6	C_6_H_10_O	769.5	764	1.05 ± 0.12	0.16 ± 0.04		0.59 ± 0.31	0.09 ± 0.02			0.04 ± 0.01	0.02 ± 0.004			
hexanal	66-25-1	C_6_H_12_O	776	766							0.01 ± 0.002		0.04 ± 0.01			0.01 ± 0.001
(*E*)-2-hexenal	6728-26-3	C_6_H_10_O	827	819	0.74 ± 0.24			0.30 ± 0.14	1.35 ± 0.51	0.10 ± 0.02		0.31 ± 0.06	0.55 ± 0.12	0.01 ± 0.01		0.09 ± 0.02
(2*E*,4*E*)-hexadienal	142-83-6	C_6_H_8_O	878	871	0.12 ± 0.08			<0.01	0.03 ± 0.01	0.01 ± 0.01		0.02 ± 0.003	0.01 ± 0.003			
α-campholenal	4501-58-0	C_10_H_16_O	1105	1098							<0.01				0.01 ± 0.002	
decanal	112-31-2	C_10_H_20_O	1185	1178	0.15 ± 0.05	0.40 ± 0.02	0.69 ± 0.13	0.29 ± 0.13		0.02 ± 0.002	0.04 ± 0.01	0.02 ± 0.01			0.13 ± 0.01	0.09 ± 0.002
decanoyl acetaldehyde	56505-80-7	C_12_H_22_O_2_		1367	1.59 ± 0.70	2.74 ± 1.23	6.55 ± 2.56	2.96 ± 0.86			0.04 ± 0.01		0.16 ± 0.09	0.33 ± 0.08	1.54 ± 0.33	0.62 ± 0.16
***Aliphatic Ester***																
*n*-nonanyl acetate	143-13-5	C_11_H_22_O_2_	1292	1284			0.19 ± 0.19	0.05 ± 0.03								
***Aliphatic Ketones***																
sulcatone	110-93-0	C_8_H_14_O	964	956									0.03 ± 0.01	0.02 ± 0.01		0.01 ± 0.002
2-nonanone	821-55-6	C_9_H_18_O	1072	1063							<0.01				0.01 ± 0.003	0.01 ± 0.001
2-undecanone	112-12-9	C_11_H_22_O	1274	1272					0.17 ± 0.07	<0.01	5.51 ± 0.61	1.93 ± 0.56	2.67 ± 1.02	2.48 ± 0.47	22.89 ± 1.97	9.92 ± 1.02
3-dodecanone	1534-27-6	C_12_H_24_O	1370	1364							<0.01				0.02 ± 0.001	
2-dodecanone	6175-49-1	C_12_H_24_O	1375	1367					<0.01	<0.01	<0.01	<0.01	0.08 ± 0.02	<0.01	0.06 ± 0.01	0.04 ± 0.004
**Compound**	**CAS Number**	**Molecular Formula**	**RI**	**RI**	**Relative Content (%)**											
					**HS-SPME**				**Essential Oil HS-SPME**				**Essential Oil Direct Injection**			
					**Leaf**	**Stem**	**Rhizome**	**Whole**	**Leaf**	**Stem**	**Rhizome**	**Whole**	**Leaf**	**Stem**	**Rhizome**	**Whole**
2-tridecanone	593-08-8	C_13_H_26_O	1477	1466					0.06 ± 0.02	0.12 ± 0.03	0.09 ± 0.01	0.17 ± 0.02	2.66 ± 0.76	1.70 ± 0.39	1.57 ± 0.17	2.93 ± 0.41
2-pentadecanone	2345-28-0	C_15_H_30_O	1681	1671								<0.01	0.14 ± 0.04	0.05 ± 0.03		0.14 ± 0.04
***Aromatic Compounds***																
*p*-cymene	99-87-6	C_10_H_14_	1014	1010	0.03 ± 0.02	0.07 ± 0.002	0.06 ± 0.01	0.08 ± 0.04	0.03 ± 0.001	0.09 ± 0.01	0.15 ± 0.01	0.13 ± 0.02	0.04 ± 0.003	0.11 ± 0.01	0.06 ± 0.01	0.11 ± 0.003
*p*-cymenene	1195-32-0	C_10_H_12_	1073	1068			0.05 ± 0.01	0.06 ± 0.01		0.04 ± 0.01	0.02 ± 0.004					
methyl salicylate	119-36-8	C_8_H_8_O_3_	1172	1169					<0.01	0.01 ± 0.002		0.01 ± 0.001		0.05 ± 0.01		0.03 ± 0.003
***Hydrocarbon***																
nonane	111-84-2	C_9_H_20_	900	890						<0.01	0.01 ± 0.002	0.01 ± 0.002	0.01 ± 0.003	0.01 ± 0.002	0.01 ± 0.005	0.01 ± 0.001
***Monoterpenes***																
tricyclene	508-32-7	C_10_H_16_	921	914										0.01 ± 0.001	<0.01	<0.01
α-thujene	2867-05-2	C_10_H_16_	924	917			0.13 ± 0.05	0.08 ± 0.02			0.19 ± 0.02	0.06 ± 0.01		0.01 ± 0.001	0.04 ± 0.01	0.02 ± 0.001
α-pinene	80-56-8	C_10_H_16_	933	925	0.26 ± 0.04	2.08 ± 0.11	9.45 ± 0.69	4.68 ± 0.92	0.52 ± 0.03	4.78 ± 0.41	14.74 ± 1.00	7.91 ± 0.72	0.44 ± 0.01	2.04 ± 0.59	7.28 ± 0.76	3.90 ± 0.51
α-fenchene	471-84-1	C_10_H_16_	944	938											<0.01	
camphene	79-92-5	C_10_H_16_	946	940	0.13 ± 0.01	0.73 ± 0.03	0.78 ± 0.25	0.73 ± 0.10	0.31 ± 0.01	2.06 ± 0.25	1.53 ± 0.11	1.10 ± 0.07	0.22 ± 0.01	1.00 ± 0.31	0.77 ± 0.08	0.55 ± 0.06
sabinene	3387-41-5	C_10_H_16_	967	963		0.14 ± 0.02	2.07 ± 0.23	0.40 ± 0.09						0.02 ± 0.002	0.20 ± 0.34	0.19 ± 0.04
β-pinene	127-91-3	C_10_H_16_	972	965	0.13 ± 0.01	1.98 ± 0.33	21.01 ± 2.32	9.79 ± 2.11	0.05 ± 0.01	0.94 ± 0.12	37.61 ± 1.16	6.70 ± 0.46	0.16 ± 0.004	1.39 ± 0.17	19.16 ± 2.07	8.95 ± 0.57
β-myrcene	123-35-3	C_10_H_16_	983	983	70.39 ± 1.85	59.81 ± 2.25	34.34 ± 1.44	59.74 ± 5.65	76.68 ± 1.24	78.00 ± 1.15	23.32 ± 1.18	62.95 ± 1.03	64.03 ± 2.07	41.59 ± 12.01	20.08 ± 1.31	41.69 ± 2.87
α-phellandrene	99-83-2	C_10_H_16_	998	992		0.06 ± 0.01	0.19 ± 0.03	0.10 ± 0.02	0.03 ± 0.002	0.22 ± 0.02		0.16 ± 0.01	0.01 ± 0.001	0.03 ± 0.02		0.05 ± 0.01
δ-3-carene	13466-78-9	C_10_H_16_	1005	1001							<0.01					
α-terpinene	99-86-5	C_10_H_16_	1010	1008		0.05 ± 0.01	0.13 ± 0.03	0.07 ± 0.01	0.03 ± 0.004	0.11 ± 0.003	0.14 ± 0.01	0.13 ± 0.01	<0.01	0.01 ± 0.01	0.04 ± 0.003	0.06 ± 0.01
limonene	138-86-3	C_10_H_16_	1023	1014	0.18 ± 0.01	1.73 ± 0.15	11.59 ± 1.01	5.12 ± 0.70	2.06 ± 0.16	3.07 ± 0.04	10.57 ± 1.18	6.13 ± 0.42	0.26 ± 0.01	2.00 ± 0.43	7.45 ± 0.61	5.48 ± 0.11
(*Z*)-β-ocimene	3338-55-4	C_10_H_16_	1028	1020	3.54 ± 0.29	0.61 ± 0.05		1.85 ± 0.25	3.99 ± 0.12	0.90 ± 0.01		1.40 ± 0.10	8.49 ± 1.21	1.71 ± 0.98		6.63 ± 0.15
(*E*)-β-ocimene	3779-61-1	C_10_H_16_	1038	1028	0.54 ± 0.10	0.41 ± 0.04	0.21 ± 0.03	0.36 ± 0.04	1.06 ± 0.25	0.48 ± 0.01	0.15 ± 0.01	0.74 ± 0.07	0.35 ± 0.09	0.16 ± 0.09	0.09 ± 0.003	0.58 ± 0.06
β-terpinene	99-84-3	C_10_H_16_	1049	1046					0.02 ± 0.001							
**Compound**	**CAS Number**	**Molecular Formula**	**RI**	**RI**	**Relative Content (%)**											
					**HS-SPME**				**Essential Oil HS-SPME**				**Essential Oil Direct Injection**			
					**Leaf**	**Stem**	**Rhizome**	**Whole**	**Leaf**	**Stem**	**Rhizome**	**Whole**	**Leaf**	**Stem**	**Rhizome**	**Whole**
γ-terpinene	99-85-4	C_10_H_16_	1050	1047			0.12 ± 0.02	0.05 ± 0.01	0.01 ± 0.001	0.05 ± 0.01	0.29 ± 0.05	0.14 ± 0.02		0.02 ± 0.01	0.19 ± 0.02	0.07 ± 0.05
α-terpinolene	586-62-9	C_10_H_16_	1079	1072	0.19 ± 0.02	0.10 ± 0.01	0.43 ± 0.01	0.21 ± 0.07	0.10 ± 0.001	0.21 ± 0.01	0.42 ± 0.08	0.29 ± 0.04		0.15 ± 0.03	0.36 ± 0.03	0.22 ± 0.03
(4*E*,6*Z*)-*allo*-ocimene	7216-56-0	C_10_H_16_	1116	1107	4.22 ± 0.44	0.60 ± 0.03	0.18 ± 0.01	2.00 ± 0.30	9.32 ± 0.33	2.31 ± 0.08	0.13 ± 0.01	4.66 ± 0.12	0.14 ± 0.003	0.05 ± 0.01	0.01 ± 0.002	0.11 ± 0.01
(4*E*,6*E*)-*allo*-ocimene	3016-19-1	C_10_H_16_	1129	1123	0.33 ± 0.07	0.17 ± 0.05	0.03 ± 0.004	0.15 ± 0.03	0.39 ± 0.003	0.17 ± 0.002	0.02 ± 0.001	0.32 ± 0.02				
***Sesquiterpenes***																
α-cubebene	17699-14-8	C_15_H_24_	1351	1348	0.06 ± 0.03				0.01 ± 0.001	<0.01		<0.01	0.05 ± 0.004			
α-copaene	3856-25-5	C_15_H_24_	1375	1377					<0.01			0.01 ± 0.001	0.04 ± 0.005	0.04 ± 0.01		0.01 ± 0.001
β-elemene	515-13-9	C_15_H_24_	1388	1386	0.19 ± 0.02		0.17 ± 0.02	0.17 ± 0.04	0.01 ± 0.001	0.02 ± 0.01	0.03 ± 0.003	0.03 ± 0.01	0.11 ± 0.01	0.14 ± 0.03	0.11 ± 0.01	0.13 ± 0.02
(*Z*)-β-caryophyllene	118-65-0	C_15_H_24_	1405	1402					<0.01	0.01 ± 0.001						
(*Z*)-α-bergamotene	18252-46-5	C_15_H_24_	1413	1406					<0.01	<0.01						
(*E*)-β-caryophyllene	87-44-5	C_15_H_24_	1419	1425	1.72 ± 0.34	1.89 ± 0.12	1.78 ± 0.27	1.70 ± 0.05	0.13 ± 0.02	0.41 ± 0.15	0.42 ± 0.04	0.32 ± 0.10	0.97 ± 0.04	2.22 ± 0.24	1.70 ± 0.22	1.56 ± 0.23
(*E*)-α-bergamotene	13474-59-4	C_15_H_24_	1432	1433	0.19 ± 0.04	0.30 ± 0.02	0.03 ± 0.002	0.08 ± 0.004	0.01 ± 0.001	0.02 ± 0.01	<0.01	0.01 ± 0.003	0.17 ± 0.02	0.18 ± 0.03	0.02 ± 0.003	0.10 ± 0.02
(*Z*)-β-farnesene	28973-97-9	C_15_H_24_	1446	1438	0.37 ± 0.05	0.34 ± 0.03	0.20 ± 0.02	0.29 ± 0.05	0.03 ± 0.001	0.08 ± 0.02	0.03 ± 0.01	0.06 ± 0.01	0.42 ± 0.01	0.77 ± 0.10	0.35 ± 0.06	0.45 ± 0.07
α-humulene	6753-98-6	C_15_H_24_	1451	1458	0.16 ± 0.03	0.29 ± 0.01		0.17 ± 0.003	0.01 ± 0.002	0.05 ± 0.02	0.04 ± 0.004	0.03 ± 0.01	0.11 ± 0.01	0.35 ± 0.06	0.27 ± 0.03	0.22 ± 0.03
alloaromadendrene	25246-27-9	C_15_H_24_	1460	1460					<0.01			0.01 ± 0.002				
(*Z*,*E*)-α-farnesene	26560-14-5	C_15_H_24_	1480	1471	0.37 ± 0.06				0.02 ± 0.002							
β-selinene	17066-67-0	C_15_H_24_	1482	1480	0.47 ± 0.04	0.83 ± 0.01	0.41 ± 0.05	0.16 ± 0.03	0.04 ± 0.003							
valencene	4630-07-3	C_15_H_24_	1484	1490	0.10 ± 0.01	0.20 ± 0.003	0.08 ± 0.01	<0.01	0.01 ± 0.001	0.03 ± 0.01	0.01 ± 0.001	0.02 ± 0.003	0.20 ± 0.03		0.10 ± 0.01	
α-selinene	473-13-2	C_15_H_24_	1490	1494	0.36 ± 0.03	0.52 ± 0.03		0.28 ± 0.02	0.03 ± 0.01	0.09 ± 0.02	0.04 ± 0.004	0.06 ± 0.01	0.44 ± 0.02		0.33 ± 0.04	
β-bisabolene	495-61-4	C_15_H_24_	1500	1499					<0.01							
γ-cadinene	39029-41-9	C_15_H_24_	1507	1508									0.05 ± 0.004	0.04 ± 0.02		
(*Z*)-calamenene	72937-55-4	C_15_H_22_	1510	1510	0.08 ± 0.03				0.01 ± 0.001	0.01 ± 0.002		0.01 ± 0.001	0.19 ± 0.02	0.17 ± 0.05		0.11 ± 0.02
δ-cadinene	483-76-1	C_15_H_24_	1515	1513	0.04 ± 0.02				<0.01	0.01 ± 0.002		0.01 ± 0.001	0.04 ± 0.004	0.08 ± 0.01		0.06 ± 0.01
cadina-1,4-diene	16728-99-7	C_15_H_24_	1525	1524					<0.01			<0.01	0.04 ± 0.001	0.04 ± 0.01		0.04 ± 0.01
**Compound**	**CAS Number**	**Molecular Formula**	**RI**	**RI**	**Relative Content (%)**											
					**HS-SPME**				**Essential Oil HS-SPME**				**Essential Oil Direct Injection**			
					**Leaf**	**Stem**	**Rhizome**	**Whole**	**Leaf**	**Stem**	**Rhizome**	**Whole**	**Leaf**	**Stem**	**Rhizome**	**Whole**
***Terpene Alcohols***																
linalool	78-70-6	C_10_H_18_O	1086	1074		0.10 ± 0.02		0.04 ± 0.003	0.11 ± 0.03	0.14 ± 0.02	0.08 ± 0.01	0.06 ± 0.01	0.23 ± 0.03	0.37 ± 0.02	0.11 ± 0.001	0.18 ± 0.01
borneol	507-70-0	C_10_H_18_O	1152	1144						0.01 ± 0.01				0.02 ± 0.004		
terpinen-4-ol	562-74-3	C_10_H_18_O	1163.5	1162			0.03 ± 0.01	0.05 ± 0.01	<0.01	0.04 ± 0.01	0.24 ± 0.05	0.13 ± 0.02			0.43 ± 0.05	0.40 ± 0.03
α-terpineol	98-55-5	C_10_H_18_O	1175	1171					0.01 ± 0.003	0.08 ± 0.02	0.15 ± 0.03	0.13 ± 0.03		0.42 ± 0.12	0.38 ± 0.04	0.51 ± 0.09
(*E*)-geraniol	106-24-1	C_10_H_18_O	1237	1231					<0.01	<0.01	<0.01	<0.01	0.04 ± 0.01	0.03 ± 0.01	<0.01	0.02 ± 0.002
perilla alcohol	536-59-4	C_10_H_18_O	1281	1273		0.10 ± 0.03		<0.01								
nerolidol	40716-66-3	C_15_H_26_O	1549	1540		0.15 ± 0.01		0.04 ± 0.001	<0.01	0.01 ± 0.001		0.01 ± 0.001	0.32 ± 0.01	1.22 ± 0.48	0.08 ± 0.01	0.26 ± 0.04
spathulenol	6750-60-3	C_15_H_24_O	1568	1569								<0.01	0.35 ± 0.02	0.66 ± 0.45		0.29 ± 0.02
globulol	51371-47-2	C_15_H_26_O	1579	1580									0.09 ± 0.01	0.19 ± 0.07		0.11 ± 0.02
veridiflorol	552-02-3	C_15_H_26_O	1580	1588									0.13 ± 0.01	0.18 ± 0.04		0.09 ± 0.02
isospathulenol	88395-46-4	C_15_H_24_O	1626	1625									0.08 ± 0.01	0.13 ± 0.05		0.05 ± 0.01
α-cadinol	481-34-5	C_15_H_26_O	1641	1642									0.09 ± 0.01	0.07 ± 0.03		0.05 ± 0.01
(2*E*,6*E*)-farnesol	106-28-5	C_15_H_26_O	1709	1700									0.04 ± 0.01	0.05 ± 0.02		0.03 ± 0.01
***Terpene Aldehydes***																
β-cyclocitral	432-25-7	C_10_H_16_O	1196	1194					0.01 ± 0.001	0.01 ± 0.004	0.01 ± 0.004	0.01 ± 0.002	0.02 ± 0.004	0.04 ± 0.004	0.05 ± 0.003	0.03 ± 0.003
geranial	141-27-5	C_10_H_16_O	1247	1242							<0.01				0.11 ± 0.02	
***Terpene Esters***																
fenchyl acetate	13851-11-1	C_12_H_20_O_2_	1214	1205						0.01 ± 0.004	0.02 ± 0.01	0.01 ± 0.002			0.05 ± 0.004	0.05 ± 0.01
linalyl acetate	115-95-7	C_12_H_20_O_2_	1241	1235	0.08 ± 0.04											
bornyl acetate	76-49-3	C_12_H_20_O_2_	1270	1271	0.60 ± 0.08	3.12 ± 0.05	3.37 ± 0.43	1.91 ± 0.39	0.49 ± 0.20	3.29 ± 0.96	2.62 ± 0.62	1.36 ± 0.29	1.79 ± 0.18	14.55 ± 1.61	5.56 ± 0.10	4.15 ± 0.18
α-terpinyl acetate	80-26-2	C_12_H_20_O_2_	1333	1330			0.31 ± 0.03				0.07 ± 0.02					
neryl acetate	141-12-8	C_12_H_20_O_2_	1343	1333			0.13 ± 0.01				0.03 ± 0.01		0.09 ± 0.01	0.30 ± 0.04	0.15 ± 0.004	0.12 ± 0.01
geranyl acetate	105-87-3	C_12_H_20_O_2_	1361	1348	0.33 ± 0.05	0.46 ± 0.03	0.48 ± 0.03	0.27 ± 0.03	0.08 ± 0.02	0.18 ± 0.03	0.10 ± 0.03	0.18 ± 0.03		3.11 ± 0.19	0.96 ± 0.52	
**Compound**	**CAS Number**	**Molecular Formula**	**RI**	**RI**	**Relative Content (%)**											
					**HS-SPME**				**Essential Oil HS-SPME**				**Essential Oil Direct Injection**			
					**Leaf**	**Stem**	**Rhizome**	**Whole**	**Leaf**	**Stem**	**Rhizome**	**Whole**	**Leaf**	**Stem**	**Rhizome**	**Whole**
(2*E*,6*E*)-farnesyl acetate	4128-17-0	C_17_H_28_O_2_	1816	1803											0.01 ± 0.01	
***Terpene Ketones***																
pinocarvone	30460-92-5	C_10_H_14_O	1140	1137						<0.01	0.02 ± 0.01			0.03 ± 0.01	0.03 ± 0.004	0.03 ± 0.001
carvone	99-49-0	C_10_H_14_O	1218	1215							<0.01				0.03 ± 0.003	
piperitone	89-81-6	C_10_H_16_O	1232.5	1228						<0.01	<0.01			0.06 ± 0.03	0.02 ± 0.002	
β-damascenone	23726-93-4	C_13_H_18_O	1361	1361					0.01 ± 0.001			0.01 ± 0.001	0.09 ± 0.02			0.64 ± 0.23
***Terpene Oxides***																
(*E*)-limonene oxide	4959-35-7	C_10_H_16_O	1124	1115							<0.01				0.01 ± 0.001	
caryophyllene oxide	1139-30-6	C_15_H_24_O	1573	1576		0.11 ± 0.005		0.03 ± 0.005			<0.01		0.27 ± 0.04	0.66 ± 0.42	0.27 ± 0.05	0.25 ± 0.02

^a^ The literature retention indices were obtained from [22,23,24,25,26]; ^b^ retention indices, using *n*-paraffin (C_5_–C_25_) as references; ^c^ values are mean ± SD of triplicates; and ^d^ references the whole plant.

**Table 2:** Concentrations of chemical groups from different parts of *H. cordata* as analyzed by different extractions.

Compound	Relative Content (%) ^a^											
	HS-SPME				Essential Oil HS-SPME				Essential Oil Direct Injection			
	Leaf	Stem	Rhizome	Whole ^b^	Leaf	Stem	Rhizome	Whole	Leaf	Stem	Rhizome	Whole
*****Aliphatic alcohols*****	5.14			0.70	0.75	0.10	0.09	0.30	0.12	0.16	0.44	0.28
*****Aliphatic aldehydes*****	3.65	3.30	7.24	4.14	1.47	0.13	0.09	0.39	0.78	0.34	1.68	0.81
*****Aliphatic ester*****			0.19	0.05								
*****Aliphatic ketones*****					0.23	0.12	5.60	2.10	5.58	4.25	24.55	13.05
*****Aromatic compounds*****	0.03	0.07	0.11	0.13	0.03	0.14	0.17	0.14	0.04	0.16	0.06	0.14
*****Hydrocarbon*****						<0.01	0.01	0.01	0.01	0.01	0.01	0.01
*****Monoterpenes*****	79.91	68.47	80.66	85.33	94.57	93.30	89.11	92.69	74.10	50.19	55.67	68.50
*****Sesquiterpenes*****	4.27	4.69	2.79	3.00	0.32	0.78	0.59	0.59	3.09	4.65	3.10	2.95
*****Terpene alcohols*****		0.35	0.03	0.13	0.12	0.28	0.47	0.33	1.37	3.34	1.00	1.99
*****Terpene aldehydes*****					0.01	0.01	0.01	0.01	0.02	0.04	0.17	0.03
*****Terpene esters*****	1.01	3.58	4.29	2.18	0.57	3.48	2.84	1.55	1.88	17.96	6.73	4.32
*****Terpene ketones*****					0.01		0.02	0.01	0.09	0.09	0.08	0.67
*****Terpene oxides*****		0.11		0.03			<0.01		0.27	0.66	0.28	0.25

^a^ Values are the means of triplicates, and ^b^ references the whole plant.

## Data Availability

Data sharing not applicable. No new data were created or analyzed in this study. Data sharing is not applicable to this article.

## Sample Availability

Samples of the compounds are not available from the authors.
